# Small Animal Veterinarians’ Perceptions, Experiences, and Views of Common Dog Breeds, Dog Aggression, and Breed-Specific Laws in the United States

**DOI:** 10.3390/ijerph16214081

**Published:** 2019-10-23

**Authors:** Lori R. Kogan, Regina M. Schoenfeld-Tacher, Peter W. Hellyer, James A. Oxley, Mark Rishniw

**Affiliations:** 1 Department of Clinical Sciences, College of Veterinary Medicine and Biomedical Sciences, Colorado State University, Fort Collins, CO 80523, USA; Peter.Hellyer@ColoState.EDU; 2 Department of Molecular Biomedical Sciences, College of Veterinary Medicine, North Carolina State University, Raleigh, NC 27607, USA; rmschoen@ncsu.edu; 3 Measham, 102 Bosworth Road, Measham, Swadlincote DE12 7Q, UK; james_oxley1@hotmail.com; 4 Veterinary Information Network, 777 W Covell Blvd, Davis, CA 95616, USA; mrishniw@vin.com

**Keywords:** canine, bite, aggression, pit bull, breed, public health

## Abstract

Dog aggression directed towards humans is a common and serious behavioral and public health issue. This cross-sectional study was designed to gain insights into U.S. small animal veterinarians’ views and experiences with the most common dog breeds in the U.S., dog aggression, and breed-specific legislation. An electronic survey was distributed via email to an online veterinary community, and responses were summarized and compared by means of χ^2^ and Cochran–Mantel–Haenszel tests. Most respondents were concerned about the risks of dog bite injuries, but the majority were not in favor of banning specific breeds of dogs in order to enhance public safety. When participants rated the perceived bite risk associated with popular dog breeds, Chow Chows were perceived as the highest risk, with pit bull types categorized as a moderate risk. Golden Retrievers were seen as the most appropriate for families with children. Public education about animal behavior was the most frequently endorsed policy intervention to increase public safety. These findings suggest that most veterinarians feel that banning an entire dog breed is not an effective way to ensure human safety. Instead, most respondents endorsed alternative initiatives, such as public education and stricter leash laws, to reduce the risk of dog bites.

## 1. Introduction

Dog ownership is common across the world, yet the United States has more dogs per person than any other country, with 38.4% of U.S. households owning a dog [1]. Dog ownership has been linked with improved physical and psychological health outcomes and overall wellbeing, but also zoonotic, injury, and bite risks [2,3,4,5]. Human directed dog aggression is a common and serious public health issue with a multi-factorial etiology [6,7,8,9]. Dog bites in the U.S. are one of the top categories of nonfatal injuries for children aged 5–9 years [10] with even higher numbers in developing regions of the world [4]. Including children and adults, there are an estimated 108 dog bites per 100,000 people in the U.S. [11], with much more unreported [12].

In response to this public health risk, many countries and U.S. cities have introduced breed-specific legislation (e.g., the banning or restriction of specific breeds) [13,14,15,16,17,18,19,20]. The pit bull (actually consisting, in the U.S., of three separate breeds—American Staffordshire Terrier, Staffordshire Bull Terrier, and American Pit Bull Terrier) is a commonly banned breed. Specific banned breeds vary by country and U.S. states and cities [21,22,23]. Given the prevalence of breed-specific legislation, there is a surprising lack of evidence to support its efficacy. This is partially due to the large numbers of mixed breed dogs; estimated in the U.S. to be 51% of all dogs. In most cases, identification of mixed breed dogs is based on physical appearance, creating the potential for misidentification or generalization when bites occur [24,25]. As noted by Scott and Fuller [26], physical appearance is a poor indicator of breed or behavior when dealing with mixed breed dogs. Several studies investigating bite reports have reported similar inaccuracies, regardless of the level of prior knowledge of the person performing the identification. Members of the general public, animal shelter workers, law enforcement officers, and human health care professionals have all been shown to make incorrect breed determinations [27,28,29,30,31,32,33]. The media also plays a role in shaping the public opinion in relation to specific breeds, yet media reports are often inaccurate and subjective [34,35,36]. Podberscek [37], for example, reviewed UK newspaper articles both before and after the implementation of the UK Dangerous Dogs Act of 1991 and suggested that the media could negatively influence the public perception of specific dog breeds without adequate, accurate information. These inaccuracies are part of the reason why the CDC (Centers for Disease Control and Prevention), in 1998, stopped collecting breed data in dog-attack fatalities [13]. 

Even though there is increasing evidence that suggests that breed bans do not decrease dog bites, more countries and communities are passing breed ban legislation. In Ireland, for example, the percent of dog bites requiring hospitalization has increased by 21% (adjusted for population growth) since implementing the restrictions of 13 breeds in 1998 [38]. Studies in Toronto and Ontario, Canada, fail to show positive results from pit bull breed bans [39]. Many professional organizations, such as the British Veterinary Association, The Federation of Veterinarians of Europe, New Zealand Veterinary Association and American Veterinary Medical Association have taken a public stance against breed-specific legislation [40,41,42], in part due to the growing evidence that suggests that breed ban legislation has negative implications for dog welfare [15,43,44]. This has led countries such as the Netherlands to lift their breed ban because it was deemed ineffective [13]. 

Notwithstanding the existing research on dog aggression and breed-specific legislation, there has been little research conducted on veterinarians’ perceptions of these topics. Therefore, the objective of this study was to gain insights into US small animal veterinarians’ views and experiences with the most common dog breeds in the US, dog aggression, and breed-specific legislation. 

## 2. Materials and Methods 

In collaboration with VIN (Veterinary Information Network—an online veterinary community), an anonymous online survey was created to evaluate veterinarians’ views regarding common dog breeds. This survey was administered directly through the VIN data collection portal. The survey was created and tested by researchers at Colorado State University and VIN. As part of this development, a pilot test in which the survey was distributed to a small sample of VIN members (~10) was conducted to screen for question flow, appropriate branching, ambiguous questions, and survey errors. Feedback from the pilot test was analyzed and incorporated into the final version of the survey. A link to the survey was distributed via an email invitation to all VIN members (n ~ 35,000), and access was made available from 18 June 2018–14 August 2018. A follow-up message was sent two weeks after the initial invitation. Only data from respondents who stated they currently practice veterinary medicine as a small animal practitioner in the U.S. were included in the study. This was an anonymous survey; therefore, written informed consent was not required; instead, an introductory statement included an explanation of the study and the fact that potential participants were giving implied consent by completing the survey. The study was categorized as exempt by the Colorado State University’s Institutional Review Board.

### Questionnaire Design 

The questionnaire consisted of nine sections, beginning with an introduction that explained the purpose of the study. This was followed by a section gathering respondents’ demographic information (age, gender, and number of children). The second section asked about current dog ownership, breed bans within the respondent’s locality, and their consideration of a pit bull breed dog as a pet. 

Section three presented questions relating to whether the respondent was currently a practicing veterinarian, veterinary practice type (e.g., private, not for profit), main practice type (e.g., small animal, large animal, animal shelter, or feline-only), their number of years in veterinary practice, and their experiences with pit bull dogs inside and outside of the veterinary practice. 

The fourth section included 18 statements and asked participants to indicate their level of agreement (i.e., strongly disagree, disagree, neither agree/disagree, agree, strongly agree), with each statement. These questions pertained to general dog ownership (e.g., “*an adult should be able to own any breed of dog*”); veterinarians involvement in the breed ban legislation (e.g., “*veterinarians should support breed ban legislation in their local communities*”); banning breeds and authority involvement (e.g., “*banning certain breeds of dog by a government entity (city, county, state) is an overreach of governmental authority*”); and perceptions of banned breed ownership and public safety (e.g., “*banning specific breeds improves public safety*”). 

The next section included three questions related to a list of twenty-three dog breeds. These breeds, with the exception of pit bull, were selected to reflect the most common dog breeds, according to the American Kennel Association, in the U.S. [45]. Pit bull was included in the list, even though it is not an actual breed, because of the common use of the term. The first question asked respondents to rate each breed in terms of serious bite risk from ‘minimal’, ‘moderate’, ‘high risk’, or ‘don’t know’. A further two questions asked participants if they feel some breeds should be banned and an open-ended question asking them to explain the reason for their answer. The next question asked participants to “Picture you are out for a walk and the following adult dog off-leash runs up to you. Please indicate your immediate emotional response” for a list of 23 common breeds. The possible answers ranged from 0 = petrified to 10 = deliriously happy/excited. The following question asked participants to indicate whether they thought each dog breed was appropriate for families with children under the age of 18. The last section asked about participants’ endorsement to several dog-related laws that might affect public safety (e.g., “*Stricter leash laws”, “Mandatory muzzling of specific breeds when in public*”). 

Statistical analysis, including descriptive statistics, Chi-square, and Cochran–Mantel–Haenszel test (CMH), was conducted with a commercially available statistical software program (SPSS, Version 25; IBM Corp, Armonk, NY, USA). The CMH was used to test the association between gender and outcomes to the agreement level statements (agree or disagree) while taking into account the stratification of child status and pit bull ownership. The agreement statements were recoded into binary statements whereby ‘strongly agree’ and ‘agree’ were combined into ‘agree’ and ‘neutral’, ‘disagree’ and ‘strongly disagree’ were recoded into ‘disagree’. CMH is used in observational studies to measure confounding variables when random assignment is not possible [46]. Due to the number of analyses conducted, the significance level (α) was set at a more conservative level of *p* = 0.01. Not all survey questions received responses; therefore, the number corresponding to each particular question is indicated in the text and the tables.

## 3. Results

A total of 1961 surveys were completed by U.S. veterinarians providing care to small animals. This sample consisted of primarily small animal practitioners (*n* = 1637, 83.5%), but also included others who work in small animal/exotic practices (*n* = 137, 7.0%), mixed animal practices (*n* = 54, 2.8%), and those working in animal shelters (*n* = 29, 1.5%); (other or not applicable (NA), *n* = 104, 5.3%). When asked how long they have been in practice (*n* = 1939), the mean was 17.5 years (SD 12.6) with a median of 16 years. 

The respondents included 1497 (76.3%) females and 439 (22.4%) males, while 25 (1.3%) respondents chose other or NA. The number of respondents (*n* = 1961) who reported having children was 947 (48.3%) with 1014 (51.7%) reporting no children. The percent of females with children (*n* = 631; 42.2%) and the percent of males with children (*n* = 306; 69.7%) was statistically different (χ^2^ = 103.19, *df* = 1, *p* < 0.001). The respondents’ age (*n* = 1950) included 204 (10.5%) under 30 years of age, 509 (26.1%) whose ages were 30–39, 433 (22.2%) whose ages were 40–49, 452 (23.2%) whose ages were 50–59, and 352 (18.1%) whose ages were 60 or older. 

The sample consisted of 1625 (82.9%) current dog owners and 336 (17.2%) participants who did not own a dog at the time of the survey. Among the dog owners who answered a question about their dog’s breed (*n* = 1618), 348 (21.5%) reported owning a pit bull-type dog, and 1270 (78.5%) reported owning a breed other than a pit bull. The percent of females owning pit bulls (*n* = 290; 23.2%) and males owning pit bulls (*n* = 53; 15.1%) was statistically different (χ^2^ = 10.58, *df* = 1, *p* = 0.001). The participants who reported owning a dog, but not a pit bull, were asked if they would consider a pit bull as a pet (*n* = 1270), to which 837 (65.9%) said yes and 433 (34.1%) said no. 

When asked about their experiences with pit bulls, only two (out of 1929) people reported not ever having had any direct interactions with pit bull dogs at work. The majority of participants, when asked about their experiences with pit bull dogs at work (*n* = 1925), reported these experiences as positive: very positive (643, 33.4%), positive (930, 48.3%), neutral (304, 15.8%), negative (44, 2.3%) and very negative (4, 0.2%). When asked about their experiences with pit bulls outside of work (*n* = 1957), 1792 (91.6%) reported having at least some experience with pit bulls outside of work, with only (137, 7.0%) reporting no experience, and (28, 1.4%) said they did not remember). When asked about these experiences (*n* = 1792), most reported very positive (487, 27.2%) or positive (860, 48.0%), with fewer reporting neutral (338, 18.9%), negative (81, 4.5%), or very negative (26, 1.5%). 

### 3.1. Dog Aggression

Participants were next asked four questions about dog aggression (Table 1). The majority of respondents reporting feeling that dog bites are a serious public health issue (81.1%), and that dog aggression against other dogs is a serious community/societal problem (78.0%). Most also agreed that owners of aggressive/dangerous dogs should be held legally accountable if their dog attacks/bites another dog (95.2%) or a person (97.2%). The Cochran–Mantel–Haenszel test was used to assess differences in responses to the aggression questions based on gender, controlling for child status, and pit bull ownership, and no differences were found between men and women for any of the four dog aggression questions. 

### 3.2. Breed Characteristics

The next four questions pertained to breed characteristics (Table 2). The majority of respondents reported feeling that some breeds of dogs are more likely to be aggressive towards other dogs (70.1%) or people (65.0%) than other breeds and that certain breeds of dogs are better suited to working with trained handlers (e.g., in law enforcement or the military) than as family pets (57.5%). Yet, most respondents (65.4%) agreed that “Depending on the circumstances, all breeds of dogs are equally likely to bite a person”.

When asked if “some breeds of dogs are more likely to be aggressive towards other dogs than other breeds”, males and females with children and no pit bull differed in agreement level (χ^2^ = 10.57; *df* 1, *p* < 0.001); 197 (87.6%) males agreed compared to 342 (77.0%) females. The same results were found for the question “Some breeds of dogs are more likely to be aggressive towards people than other breeds” whereby there was a gender difference for those with children and no pit bull (χ^2^ = 9.76; *df* 1, *p* = 0.002); 188 (83.6%) males agreed compared to 322 (72.7%) females. Similarly, the question “Depending on the circumstances, all breeds of dogs are equally likely to bite a person”, there was a difference between men and women with children and no pit bull (Pearson χ^2^ = 17.36; *df* 1, *p* < 0.001); 100 (44.2%) males agreed compared to 271 (61.2%) females. No differences in the agreement level were found for the statement, “Certain breeds of dogs are better suited to working with trained handlers (e.g., in law enforcement or the military) than as family pets”. 

### 3.3. Breed Banning

Participants were next asked their views about specific breed banning (Table 3). Overall, participants were opposed to breed bans. The majority (75.3%) agreed that banning specific breeds creates an animal welfare issue and that banning certain breeds of dogs by a government entity (city, county, state) is an overreach of governmental authority (76.2%). Most disagreed that particular dog breeds should be banned from being around children (87.8%) or that banning specific breeds of dogs improves public safety (89.2%). 

Significant gender differences in the agreement level to the statement, “Banning specific breeds creates an animal welfare issue” for participants with children and no pit bull were found (Pearson *χ*^2^ = 18.50; *df* 1, *p* < 0.001); 124 (55.6%) males agreed compared to 318 (72.3%) females. Gender also made a difference for those with no children and did own a pit bull (Pearson *χ*^2^ = 11.05; *df* 1, *p* = 0.004); 22 (78.6%) males agreed compared to 180 (94.2%) females in agreement level to the statement “Banning certain breeds of dogs by a government entity (city, county, state) is an overreach of governmental authority”. When asked if “Some dog breeds should be banned from being around children”, gender differences were found for those with or without children who did not own a pit bull. For those with children, 60 (26.9%) males agreed compared to 56 (12.6%) females. (Pearson *χ*^2^ = 20.98; *df* 1, *p* < 0.001). For those without children, 14 (19.7%) males agreed compared to 38 (7.4%) females (Pearson *χ*^2^ = 11.61; *df* 1, *p* = 0.001). Lastly, gender made a difference for those with children who did not own a pit bull when asked if “Banning specific breeds of dogs improves public safety”, whereby 52 (23.1%) males agreed compared to 52 (11.8%) females (Pearson *χ*^2^ = 14.59; *df* 1, *p* < 0.001).

### 3.4. Pet Ownership 

The next three questions pertained to pet and specifically dog ownership in general (Table 4). The vast majority of participants agreed that “Socially irresponsible pet ownership is a significant societal problem” (95.1%). When asked if “owning a dog is a right rather than a privilege”, the majority disagreed (81.3%). Opinions were mixed, however, on whether “any adult should be able to own any breed of dog”, 44.3% agreed while 55.7% disagreed. 

When asked if “Owning a dog is a right rather than a privilege”, gender made a difference for those with children who did not own a pit bull: 62 (27.6%) males agreed compared to 68 (15.3%) females (Pearson *χ*^2^ = 14.18; *df* 1, *p* < 0.001). No agreement level differences were found for the statements “Socially irresponsible pet ownership is a significant societal problem” or “Any adult should be able to own any breed of dog”. 

### 3.5. Veterinarians’ Role 

Veterinarians’ role as it relates to aggressive dogs and breed bans constituted the subject of the next three questions (Table 5). Most participants reported feeling that veterinarians have a role in advising clients on how to train/manage aggressive/dangerous dogs (86.3%). They did not feel that veterinarians should support breed ban legislation in their local communities (81.8%), and in fact, most (66.1%) felt that veterinarians openly supporting any type of breed ban negatively affects the public perception of veterinarians. 

There was a difference between agreement level of males and females with both children and a pit bull to the statement “Veterinarians have a role in advising clients on how to train/manage aggressive/dangerous dogs”, where 16 (64.0%) males agreed to the statement compared to 88 (90.7%) females (Pearson *χ*^2^ = 11.29; *df* 1, *p* = 0.001).

There was also a difference in agreement level between males and females with children, but no pit bulls to the statement, “Veterinarians should support breed ban legislation in their local communities” 26 (11.6%) males agreed compared to 18 (4.1%) females (Pearson *χ*^2^ = 13.74 *df* 1, *p* < 0.001)). Similarly, there was a difference between males and females with children but no pit bull to agreement with the sentiment “I feel it negatively affects the public perception of a veterinarian to openly support any type of breed ban”; 119 (52.9 %) males agreed compared to 287 (65.1%) females (Pearson *χ*^2^ = 9.30; *df* 1, *p* = 0.002). 

### 3.6. Serious Bite Risk Based on Breed 

Participants were presented with a list of common breeds and asked to indicate their perception of the different breeds’ serious bite risk (defined as requiring medical treatment) as minimal, moderate, or high (or don’t know). These breeds are listed in Table 6, in order of perceived severity of the risk. The breeds felt to pose the highest risk of serious bites include Chow Chows (perceived as high risk by 60.6%), followed by Chihuahuas (perceived as high risk by 48.5%), German shepherds (47.3%), Rottweilers (44.2%), Akitas (41.8%), and Belgian Malinois (39.7%). The dog breeds with the lowest perceived risk of serious bites include Golden retriever (4.6%), Labrador retriever (4.6%), English bulldog (4.1%), Standard poodle (3.6%), and Beagles (2.3%) had the lowest perceived risk. 

### 3.7. Appropriate Breeds for Families with Children 

Participants were asked how suitable they felt a list of common U.S. dog breeds are for families with children under the age of 18 with possible responses, including appropriate, neutral, inappropriate, and don’t know. These results are listed in order of decreasing level of appropriateness in Table 7. The breeds felt to be most appropriate for families with children include Golden retrievers (rated as appropriate by 76.9%), Labrador retrievers (75.7%), Beagles (67.7%), Standard poodles (62.7%), and Boxers (58.5%). The breeds felt to be the least appropriate include Chow Chows (10.3%), Akitas (10.4%), Belgian Malinois (12.2%), and Rottweilers (17.0%). 

### 3.8. Reaction to Unfamiliar Dogs Off-Leash

Participants were asked to imagine that they were out for a walk, and an adult dog, off-leash, runs up to them. They were asked to report their immediate emotional response on a scale from 0–10, with 0 = petrified and 10 = deliriously happy/excited. The mean, median, and mode scores for each breed are listed in Table 8. The breeds that were reported to elicit the most fearful/negative response were Chow Chows, Rottweilers, Akitas, German shepherds, and Belgian Malinois. Participants reported they would feel the happiest or most excited by Golden retrievers, Labrador retrievers, Beagles, and Standard poodles.

Lastly, participants were asked to indicate their degree of endorsement of several policies communities have enacted in an effort to increase public safety (*n* = 1961). These statements are listed in order of decreasing endorsement rate in Table 9. The most commonly endorsed policies include public education about animal behavior (endorsed by 90.0%), stricter leash laws (79.9%), and public education about animal welfare (78.4%).

## 4. Discussion

Dog bites and dog aggression are serious public health concerns [47,48,49,50,51]. The aim of this study was to investigate U.S. veterinarians’ experiences, perceptions, and views of dog breeds, dog aggression, breed-specific legislation. When there were significant differences in participants’ responses based on their gender, controlling for parental status and pit bull ownership, males were more likely to report feeling that some breeds are more aggressive than other breeds and were more open to some aspects of breed-specific legislation. Further study is needed to explore these gender differences to better understand the relationship between gender and parental status on veterinarians’ views on aggressive dogs, and pit bulls in particular. 

Many of the breeds identified by this study’s participants as high bite risk or inappropriate for families with young children have been named in other studies [52,53,54,55,56,57]. Yet, as Wake [58] notes, a corresponding increase in bite reports pertaining to a specific breed can follow an increase in popularity of that breed. In other words, when there are more dogs of a particular breed, this could potentially lead to an increase in reported bites by that breed, something that should be taken into consideration when attempting to collect data on breed aggression risks [58]. Although the results of this study suggest that veterinarians think some dog breeds pose a more serious bite risk than other breeds, the majority of respondents also reported feeling that, depending on the circumstances, all dog breeds are capable of biting; a sentiment echoed in numerous other papers [6,59,60]. Additional dog-specific factors such as sex and age, early experience, socialization and training, health (medical and behavioral), and reproductive status, as well as human factors, including the use of physical punishment, supervision, owner personality, and victim behavior have also been shown to influence aggressive behaviors [6,61,62,63].

Regardless of the breed, respondents overwhelmingly reported feeling that dog aggression (against both people and other dogs) is a serious public health concern and that owners of aggressive dogs should be held accountable. Importantly, participants echoed other study results in their sentiments that veterinarians should play an active role in advising clients about how to handle aggressive dogs [64,65]. Unfortunately, many veterinarians do not feel comfortable discussing aggressive behavior problems with clients [66]. For these reasons, it has been suggested that more animal behavior training is needed in veterinary curricula [66,67,68]. 

While participants acknowledge dog aggression as a serious public health concern, they do not feel that banning a specific breed is a viable solution. Some of the reasoning behind these views could be attributed to inherent problems with breed ban legislation, especially as it relates to pit bulls. As noted earlier, a central issue is that pit bull is not actually a breed, but instead a group of breeds [32]. The classification of ‘pit bull’ is typically based on a dogs’ physical resemblance to one of several breeds that have been associated with the term pit bull [28,69], when, in fact, most people are unable to identify a pit bull accurately [27,28,30,31]. 

Breed bans are often a reaction to horrific dog bite stories depicted in popular media [37,70]. Yet, the accuracy of bite reports and resultant media stories have been questioned [33]. In particular, pit bulls are often identified as problematic and dangerous [71], but in many medical reports, the breed of the offending dog is either not reported or assumed based on phenotypic characteristics [25]. In fact, one study [72] found that over one-half of dog-bite medical reports for children were missing breed information. This lack of data can lead to selection bias and compromised validity [72]. Another study found that approximately 51% of the dogs in the US are of mixed breed [73], yet the vast majority of dog bite reports include only one breed, giving the impression of a purebred [69]. Based on these studies and numerous others, it appears likely that many media reports on dog bites are misleading at best, or at worst, inaccurate. Unfortunately, however, these reports are the foundation for many dog bite and aggression studies. The CDC’s report Dog Bite-Related Fatalities From 1979–1988, for example, relied on media stories in which breed identification was based on physical appearance [74]. These reports, therefore, often demonized pit bulls by perpetuating stereotypes or false information about the breed [75,76,77]. 

In addition to doubts about the effectiveness of breed bans, their impact on animal welfare is of growing concern. Several studies have suggested that these laws are ineffective. Additionally, they are expensive, difficult to implement, and subject responsible, law-abiding dog owners to unnecessary regulations/hardships. These breed bans also negatively impact dog welfare [15,38,43,78,79,80]. It is, therefore, encouraging to note that most veterinarians, arguably a powerful lobbying group, agree with the sentiment that banning an entire dog breed is not an effective way to ensure human safety [75]. The American Veterinary Medical Association (AVMA) succinctly stated the problem with breed-specific legislation, “*While breed is a factor, the impact of other factors relating to the individual animal (such as training method, sex, and neutering status), the target (e.g., owner versus stranger), and the context in which the dog is kept (e.g., urban versus rural) prevent breed from having significant predictive value in its own right*.” [57] (p. 3).

Several studies suggest there are better solutions for dog bites and dog aggression, including better owner and public education about dog behavior and dog bite prevention, responsible ownership, and active enforcement of dog control ordinances—all of which were endorsed by the majority of participants [57,81]. Teaching adults and children how to understand dog signaling and behaviors, for example, has been found to be an effective strategy in increasing dog behavior knowledge and improving appropriate and safe behaviors of people around dogs [4,82,83]. In addition, to educate about dog behavior, there is a need for public education on pet care and welfare. Given the fact that most dog bites are from their own pet dog, educating families (e.g., children, adults, and elderly) about animal behavior has been seen as critical in reducing dog bites. Dissemination of this information could be done in a variety of ways by different community entities. Examples include information provided by veterinarians and animal-related companies (e.g., pet supplies) or distributed by city entities (e.g., utility companies, city/county news, etc.). Another consideration is the mandatory inclusion of training for owners who are ticketed or cited for a dog-related incident [84]. 

This study was not without limitations. The survey was distributed online, and respondents might not be representative of all small animal U.S. practitioners. The results should be interpreted with care and cannot be generalized to the general population for several reasons. First and foremost, it is not known if these views are shared by non-veterinarians. Additionally, this sample was predominately female and included a significant number of pit bull owners. The reasons for this are unclear. It is not known if veterinarians are more likely to own pit bulls, or if those that own pit bulls were more likely to complete this survey. These factors speak to the need for further study with the general population to assess potential differences of views and attitudes and warrant some caution when interpreting the data. In summary, veterinarians have a vested interest in a reduction of dog bite incidents within their communities. By increasing behavior training in veterinary curricula, and offering more behavior-related continuing education, it is the hope that veterinarians will feel more comfortable addressing animal aggression issues with their clients. In addition, community supported public education on dog behavior, enhanced licensure compliance, stricter leash laws, and penalties for negligent dog owners of aggressive dogs appear to be viable options to mitigate the risk of aggressive dogs that are supported by both veterinarians and current research. 

## Figures and Tables

**Table 1:** Dog aggression statements.

Statements	Agree *n* (%)	Disagree *n* (%)
Dog aggression against other dogs is a serious community/societal problem (*n* = 1952)	1523 (78.0%)	429 (22.0%)
Dog bites are a serious public health issue (*n* = 1947)	1579 (81.1%)	368 (18.9%)
Owners of aggressive/dangerous dogs should be held legally accountable if their dog attacks/bites another dog (*n* = 1950)	1856 (95.2%)	94 (4.8%)
Owners of aggressive/dangerous dogs should be held legally accountable if their dog attacks a person (*n* = 1946)	1892 (97.2%)	54 (2.8%)

**Table 2:** Perceived association between dog breed and behavior.

Statements	Agree *n* (%)	Disagree *n* (%)
Certain breeds of dogs are better suited to working with trained handlers (e.g., in law enforcement or the military) than as family pets (*n* = 1948)	1120 (57.5%)	828 (42.5%)
Some breeds of dogs are more likely to be aggressive towards other dogs than other breeds (*n* = 1952)	1369 (70.1%)	583 (29.9%)
Some breeds of dogs are more likely to be aggressive towards people than other breeds (*n* = 1954)	1271 (65.0%)	683 (35.0%)
Depending on the circumstances, all breeds of dogs are equally likely to bite a person (*n* = 1953)	1278 (65.4%)	675 (34.6%)

**Table 3:** Perceptions of breed ban legislation.

Statements	Agree *n* (%)	Disagree *n* (%)
Banning specific breeds creates an animal welfare issue (*n* = 1945)	1464 (75.3%)	481 (24.7%)
Banning certain breeds of dogs by a government entity (city, county, state) is an overreach of governmental authority (*n* = 1951)	1486 (76.2%)	465 (23.8%)
Some dog breeds should be banned from being around children (*n* = 1949)	237 (12.2%)	1712 (87.8%)
Banning specific breeds of dogs improves public safety (*n* = 1945)	210 (10.8%)	1735 (89.2%)

**Table 4:** Perceptions of pet ownership in the context of society.

Statements	Agree *n* (%)	Disagree *n* (%)
Socially irresponsible pet ownership is a significant societal problem (*n* = 1953)	1857 (95.1%)	96 (4.9%)
Any adult should be able to own any breed of dog (*n* = 1947)	862 (44.3%)	1085 (55.7%)
Owning a dog is a right rather than a privilege (*n* = 1952)	366 (18.2%)	1586 (81.3%)

**Table 5:** Perceptions of veterinary involvement with breed bans.

Statements	Agree *n* (%)	Disagree *n* (%)
Veterinarians have a role in advising clients in how to train/manage aggressive/dangerous dogs (*n* = 1949)	1682 (86.3%)	267 (13.7%)
Veterinarians should support breed ban legislation in their local communities (*n* = 1950)	87 (4.5%)	1863 (95.5%)
I feel it negatively affects the public perception of a veterinarian to openly support any type of breed ban (*n* = 1947)	1287 (66.1%)	660 (33.9%)

**Table 6:** Veterinarians’ perceptions of serious bite risk of 23 common U.S. dog breeds.

Breed	High *n* (%)	Moderate *n* (%)	Minimal *n* (%)	Don’t know *n* (%)
**High risk (> 39%)**				
Chow Chow (*n* = 1947)	1179 (60.6%)	639 (32.8%)	66 (3.4%)	63 (3.2%)
Chihuahua (*n* = 1945)	944 (48.5%)	699 (35.9%)	252 (13.0%)	50 (2.6%)
German shepherd (*n* = 1944)	920 (47.3%)	816 (42.0%)	148 (7.6%)	60 (3.1%)
Rottweiler (*n* = 1937)	856 (44.2%)	827 (42.7%)	185 (9.6%)	69 (3.6%)
Akita (*n* = 1943)	813 (41.8%)	879 (45.2%)	144 (7.4%)	107 (5.5%)
Belgian Malinois (*n* = 1944)	772 (39.7%)	819 (42.1%)	211 (10.9%)	142 (7.3%)
**Moderate risk**				
Siberian husky (*n* = 1936)	508 (26.2%)	959 (49.5%)	407 (21.0%)	62 (3.2%)
Dalmatian (*n* = 1943)	459 (23.6%)	988 (50.8%)	368 (18.9%)	128 (6.6%)
Pit bull type (*n* = 1938)	427 (22.0%)	964 (49.7%)	481 (24.8%)	66 (3.4%)
Mastiff (*n* = 1936)	350 (18.1%)	809 (41.8%)	701 (36.2%)	76 (3.9%)
Dachshund (*n* = 1944)	346 (17.8%)	1030 (53.0%)	515 (26.5%)	53 (2.7%)
Cocker spaniel (*n* = 1944)	307 (15.8%)	1050 (54.0%)	526 (27.1%)	61 (3.1%)
Jack Russell Terrier (*n* = 1940)	305 (15.7%)	1050 (54.1%)	527 (27.2%)	58 (3.0%)
Great Dane (*n* = 1945)	298 (15.3%)	810 (41.6%)	773 (39.7%)	64 (3.3%)
American bulldog (*n* = 1942)	258 (13.3%)	860 (44.3%)	745 (38.4%)	79 (41%)
Doberman pinscher (*n* = 1944)	222 (11.4%)	839 (43.2%)	820 (42.2%)	63 (3.2%)
**Low risk**				
Yorkshire terrier (*n* = 1935)	136 (7.0%)	684 (35.3%)	1056 (54.6%)	59 (3.0%)
Boxer (*n* = 1940)	104 (5.4%)	727 (37.5%)	1043 (53.8%)	66 (3.4%)
Golden retriever (*n* = 1943)	90 (4.6%)	403 (20.7%)	1393 (71.7%)	57 (2.9%)
Labrador retriever (*n* = 1938)	90 (4.6%)	473 (24.4%)	1316 (67.9%)	59 (3.0%)
English bulldog (*n* = 1944)	80 (4.1%)	609 (31.3%)	1193 (61.4%)	62 (3.2%)
Standard poodle (*n* = 1937)	69 (3.6%)	449 (23.2%)	1354 (69.9%)	65 (3.4%)
Beagle (*n* = 1939)	44 (2.3%)	573 (29.6%)	1253 (64.6%)	69 (3.6%)

**Table 7:** Veterinarians’ views on the appropriateness of ownership for families with children under the age of 18 of 23 common U.S. dog breeds.

Breed	Appropriate *n* (%)	Neutral *n* (%)	Inappropriate *n* (%)	Don’t know *n* (%)
Golden retriever (*n* = 1931)	1485 (76.9%)	390 (20.2%)	6 (0.3%)	50 (2.6%)
Labrador retriever (*n* = 1929)	1461 (75.7%)	410 (21.3%)	7 (0.4%)	51 (2.6%)
Beagle (*n* = 1933)	1309 (67.7%)	54 (29.2%)	16 (0.8%)	44 (2.3%)
Standard poodle (*n* = 1928)	1208 (62.7%)	654 (33.9%)	14 (0.7%)	52 (2.7%)
Boxer (*n* = 1934)	1132 (58.5%)	714 (36.9%)	42 (2.2%)	46 (2.4%)
English bulldog (*n* = 1931)	880 (45.6%)	897 (46.5%)	101 (5.2%)	53 (2.7%)
Yorkshire terrier (*n* = 1933)	876 (45.3%)	842 (43.6%)	166 (8.6%)	49 (2.5%)
Dachshund (*n* = 1934)	721 (37.4%)	952 (49.2%)	216 (11.2%)	45 (2.3%)
Doberman pinscher (*n* = 1932)	689 (35.7%)	1012 (52.4%)	175 (9.1%)	56 (2.9%)
Cocker spaniel (*n* = 1934)	678 (35.1%)	1018 (53.2%)	178 (9.2%)	50 (2.6%)
Great Dane (*n* = 1932)	663 (34.3%)	977 (50.6%)	242 (12.5%)	50 (2.6%)
American bulldog (*n* = 1930)	638 (33.1%)	984 (51.0%)	250 (13.0%)	58 (3.0%)
Jack Russell terrier (*n* = 1931)	634 (32.8%)	1027 (53.2%)	219 (11.3%)	51 (2.6%)
Pit bull type (*n* = 1933)	634 (32.8%)	898 (46.5%)	352 (18.2%)	49 (2.5%)
Mastiff (*n* = 1933)	554 (28.7%)	993 (51.4%)	319 (16.5%)	67 (3.5%)
Chihuahua (*n* = 1936)	437 (22.6%)	937 (48.4%)	515 (26.6%)	47 (2.4%)
Dalmatian (*n* = 1932)	412 (21.3%)	1065 (55.1%)	373 (19.3%)	82 (4.2%)
German shepherd (*n* = 1934)	409 (21.1%)	1007 (52.1%)	470 (24.3%)	48 (2.5%)
Siberian husky (*n* = 1932)	364 (18.8%)	1049 (54.3%)	467 (24.2%)	52 (2.7%)
Rottweiler (*n* = 1930)	328 (17.0%)	901 (46.7%)	648 (33.6%)	53 (2.7%)
Belgian Malinois (*n* = 1935)	237 (12.2%)	835 (43.2%)	733 (37.9%)	130 (6.7%)
Akita (*n* = 1961)	200 (10.4%)	795 (41.2%)	821 (42.5%)	114 (5.9%)
Chow Chow (*n* = 1935)	199 (10.3%)	730 (37.7%)	945 (48.8%)	61 (3.2%)

**Table 8:** Mean, median, and mode scores given by veterinarians to rate their emotional response to being approached by an unfamiliar adult dog off-leash from a list of 23 common U.S. dog breeds. (0 = petrified and 10 = deliriously happy/excited).

Breed (*n*)	Mean	Standard Deviation	Median	Mode
Chow Chow (*n* = 1619)	2.85	1.79	3	3
Rottweiler (*n* = 1592)	3.07	2.13	3	4
Akita (*n* = 1535)	3.14	1.77	3	3
German shepherd (*n* = 1577)	3.18	2.11	3	4
Belgian Malinois (*n* = 1528)	3.27	1.93	3	4
Siberian Husky (*n* = 1392)	3.93	2.00	4	4
Dalmatian (*n* = 1301)	4.23	1.89	4	4
Pit bull type (*n* = 1492)	4.31	2.62	4	4
Mastiff (*n* = 1438)	4.36	2.39	4	4
Doberman pinscher (*n* = 1410)	4.54	2.17	4	4
Chihuahua (*n* = 1294)	4.58	2.13	4	4
American bulldog (*n* = 1334)	4.67	2.30	4	4
Great Dane (*n* = 1444)	4.80	2.45	4	4
Jack Russell terrier (*n* = 1241)	5.03	1.96	5	4
Cocker spaniel (*n* = 1196)	5.18	1.91	5	4
Dachshund (*n* = 1235)	5.47	1.95	5	6
Boxer (*n* = 1312)	5.84	2.21	6	6
English bulldog (*n* = 1232)	6.02	2.11	6	6
Yorkshire terrier (*n* = 1196)	6.17	2.04	6	6
Standard poodle (*n* = 1276)	6.47	2.11	6	6
Beagle (*n* = 1235)	6.52	1.90	6	6
Labrador retriever (*n* = 1420)	7.05	2.11	7	7
Golden retriever (*n* = 1439)	7.34	2.12	8	10

**Table 9:** Veterinarians’ reported endorsement of community policies enacted in efforts to increase public safety.

Policy	Endorsement Rate *n* (%)
Public education about animal behavior	1764 (90.0%)
Stricter leash laws	1567 (79.9%)
Public education about animal welfare	1538 (78.4%)
Harsher penalties for dog owners in the event of a dog bite or attack	1419 (72.4%)
Stricter laws about picking up dog waste	1349 (68.8%)
Stricter fencing or containment laws	1261 (64.3%)
Anti-chaining & anti-tethering laws	1178 (60.1%)
Compulsory owner-dog training	863 (44.0%)
Mandatory spay/neuter for specific breeds	384 (19.6%)
Mandatory registration for specific breeds	265 (13.5%)
Mandatory muzzling of specific breeds when in public	120 (6.1%)
